# Risk Factors for Cisplatin-Induced Nephrotoxicity and Potential of Magnesium Supplementation for Renal Protection

**DOI:** 10.1371/journal.pone.0101902

**Published:** 2014-07-14

**Authors:** Yasuhiro Kidera, Hisato Kawakami, Tsutomu Sakiyama, Kunio Okamoto, Kaoru Tanaka, Masayuki Takeda, Hiroyasu Kaneda, Shin-ichi Nishina, Junji Tsurutani, Kimiko Fujiwara, Morihiro Nomura, Yuzuru Yamazoe, Yasutaka Chiba, Shozo Nishida, Takao Tamura, Kazuhiko Nakagawa

**Affiliations:** 1 Division of Pharmacotherapy, Kinki University Faculty of Pharmacy, Higashi-Osaka, Osaka, Japan; 2 Department of Pharmacy, Kinki University Faculty of Medicine, Osaka-Sayama, Osaka, Japan; 3 Department of Medical Oncology, Kinki University Faculty of Medicine, Osaka-Sayama, Osaka, Japan; 4 Division of Biostatistics, Clinical Research Center, Kinki University Faculty of Medicine, Osaka-Sayama, Osaka, Japan; H. Lee Moffitt Cancer Center & Research Institute, United States of America

## Abstract

**Background:**

Nephrotoxicity remains a problem for patients who receive cisplatin chemotherapy. We retrospectively evaluated potential risk factors for cisplatin-induced nephrotoxicity as well as the potential impact of intravenous magnesium supplementation on such toxicity.

**Patients and Methods:**

We reviewed clinical data for 401 patients who underwent chemotherapy including a high dose (≥60 mg/m^2^) of cisplatin in the first-line setting. Nephrotoxicity was defined as an increase in the serum creatinine concentration of at least grade 2 during the first course of cisplatin chemotherapy, as assessed on the basis of National Cancer Institute Common Terminology Criteria for Adverse Events version 4.0. The severity of nephrotoxicity was evaluated on the basis of the mean change in the serum creatinine level. Magnesium was administered intravenously to 67 patients (17%).

**Results:**

Cisplatin-induced nephrotoxicity was observed in 127 patients (32%). Multivariable analysis revealed that an Eastern Cooperative Oncology Group performance status of 2 (risk ratio, 1.876; P = 0.004) and the regular use of nonsteroidal anti-inflammatory drugs (NSAIDs) (risk ratio, 1.357; P = 0.047) were significantly associated with an increased risk for cisplatin nephrotoxicity, whereas intravenous magnesium supplementation was associated with a significantly reduced risk for such toxicity (risk ratio, 0.175; P = 0.0004). The development of hypomagnesemia during cisplatin treatment was significantly associated with a greater increase in serum creatinine level (P = 0.0025). Magnesium supplementation therapy was also associated with a significantly reduced severity of renal toxicity (P = 0.012).

**Conclusions:**

A relatively poor performance status and the regular use of NSAIDs were significantly associated with cisplatin-induced nephrotoxicity, although the latter association was marginal. Our findings also suggest that the ability of magnesium supplementation to protect against the renal toxicity of cisplatin warrants further investigation in a prospective trial.

## Introduction

Cisplatin (*cis*-diammine-dichloroplatinum), an inorganic platinum chemotherapeutic drug, has been widely administered either alone or in combination with other agents for the clinical treatment of various solid tumors [1]. The efficacy of cisplatin is limited, however, by severe side effects such as nephrotoxicity, neurotoxicity, ototoxicity, and emetogenicity [2], [3]. In particular, the nephrotoxicity of cisplatin is dose dependent and therefore limits the amount of drug that can be administered [4]. Procedures to reduce such toxicity include aggressive hydration with saline and simultaneous administration of mannitol, which is now accepted as the standard of care for individuals treated with regimens containing a high dose (≥60 mg/m^2^) of cisplatin [5]. Unfortunately, renal toxicity still occurs even with such hydration, highlighting the need for more effective preventive strategies.

Another approach to limiting the nephrotoxicity of cisplatin is intravenous magnesium supplementation. Cisplatin-induced nephrotoxicity is accompanied by disturbance of the renal handling of electrolytes. In particular, depletion of magnesium has emerged as a common event associated with the acute renal toxicity induced by the drug [6]. Whereas several studies have demonstrated the efficacy of magnesium supplementation for prevention of hypomagnesemia during cisplatin treatment [7]–[10], only two prospective studies, each featuring a relatively small number of patients, have evaluated its efficacy in terms of protection against cisplatin-induced nephrotoxicity [11], [12]. Despite the dearth of evidence in support of a beneficial effect of magnesium supplementation therapy on the renal toxicity of cisplatin, intravenous administration of magnesium is currently recommended for outpatients receiving high-dose cisplatin with a short hydration regimen [13]. We have therefore recently applied this procedure to all patients who receive such chemotherapy. However, given that magnesium supplementation has not been accepted as the standard of care, at least in Japan, most patients who receive high-dose cisplatin are treated with aggressive hydration in the inpatient setting.

We have now assessed a large group of unselected consecutive patients in an attempt to identify potential biological or pharmacological parameters that might predispose individuals to cisplatin-induced nephrotoxicity. We also retrospectively evaluated the potential impact of intravenous magnesium supplementation on this side effect of cisplatin treatment.

## Patients and Methods

### Eligibility criteria

We reviewed the cases in our database and retrospectively examined the clinical data of patients who received therapy including a high dose (≥60 mg/m^2^) of cisplatin in the first-line setting at the Department of Medical Oncology, Kinki University Hospital, between January 2008 and August 2012. Patients were eligible if they had pathologically confirmed malignancies and an Eastern Cooperative Oncology Group performance status (PS) of 0 to 2. Patients were excluded from the study if they had a history of cisplatin treatment or had more than one cancer. The study protocol was approved by the ethics committee of Kinki University Hospital with the condition that all data be processed and analyzed anonymously, and written informed consent was provided by all patients. The study also conforms with the provisions of the Declaration of Helsinki.

### Cisplatin administration

All regimens containing high-dose cisplatin were administered in the inpatient setting. Cisplatin was administered in 500 mL of 0.9% normal saline over 1 h. Most patients were prehydrated with 500 mL of one-quarter isotonic saline containing 5% glucose and 20 mEq of KCl, and they were posthydrated with 500 mL of 0.9% normal saline mixed with 500 mL of one-quarter isotonic saline containing 5% glucose, 20 mEq of KCl, and 10 mEq of sodium L-lactate, which was administered over 1 to 2 h and followed by 60 g of mannitol over 1 h and 20 mg of furosemide in 50 mL of 0.9% normal saline over 15 min. Antiemetic prophylaxis with 5-HT_3_ serotonin receptor antagonists plus dexamethasone was administered 15 min before the onset of chemotherapy in all cases. A neurokinin 1 (NK1) receptor antagonist was added to the antiemetic cocktail from October 2010 in response to the approval of this drug in Japan. Magnesium sulfate (20 mEq) was administered with 500 mL of one-quarter isotonic saline over 1 h after cisplatin administration as magnesium supplementation therapy to all patients from July 2011.

### Nephrotoxicity evaluation

According to a previous study [14], we adopted an increase in the serum concentration of creatinine as a measure of nephrotoxicity. The serum creatinine concentration was determined before the first course of cisplatin chemotherapy (baseline value) and weekly during chemotherapy. For evaluation of nephrotoxicity, the increase in the serum creatinine concentration was calculated as the maximum value during the first course of chemotherapy minus the baseline value. Given that the serum creatinine level is a denominator of the Cockcroft-Gault equation, changes in creatinine clearance over a short period are solely dependent on those in serum creatinine concentration. Nephrotoxicity was defined as an increase in the serum creatinine concentration of grade 2 or higher, according to the National Cancer Institute Common Terminology Criteria for Adverse Events (NCI CTCAE, version 4.0), during the first course of cisplatin chemotherapy.

### Statistical analysis

To identify risk factors potentially associated with the occurrence of a nephrotoxicity event, each factor was compared by the unpaired Student's *t* test or Fisher's exact test. Factors in the analysis included age (≥70 vs. <70 years) and PS (2 vs. 0 or 1), given that chemotherapy might be expected to result in excessive toxicity in patients with an age of ≥70 years or a PS of 2 [15]. The other factors were sex (male vs. female), tumor type, concurrent radiation treatment, hypoalbuminemia (serum albumin concentration of <3.0 g/dL), enteral or total parenteral nutrition, type 2 diabetes, hydration (≤2000 mL), intravenous magnesium supplementation, oral intake of magnesium oxide as a laxative agent, use of antihypertensive medication, treatment with an NK1 receptor antagonist, and regular use of nonsteroidal anti-inflammatory drugs (NSAIDs). The risk factors were also evaluated in multivariable analysis with the Poisson regression model. The risk ratio with 95% confidence interval (CI) was calculated for the independent prognostic factors. The mean change in serum creatinine concentration was compared between groups with the use of box-and-whisker plots showing the range (maximum and minimum), median, and quartile range (75 and 25 percentiles) and was evaluated with the unpaired Student's *t* test. Statistical analysis was performed with the use of SAS software version 9.4 (SAS Institute, Cary, NC). A *P* value of <0.05 was considered statistically significant.

## Results

### Patient characteristics

A total of 401 patients who received chemotherapy including high-dose cisplatin were eligible for the analysis. Baseline characteristics of the eligible patients are summarized in Table 1. The median age was 65 years (range, 28–80), and most patients were male (77%) and had a good PS of 0 or 1 (94%). The most common malignancies were lung cancer (36%), head and neck cancer (23%), gastric cancer (19%), and esophageal cancer (16%). Median age, sex, PS, median serum creatinine concentration at baseline, median body surface area, median body mass index, and the median dose of cisplatin in the first course of chemotherapy did not differ significantly among the types of malignancy. The various chemotherapy regimens administered to the patients are shown in Table S1.

**Table 1:** Baseline characteristics of the 401 study patients.

Characteristic			All patients	Lung cancer	Head and neck cancer	Gastric cancer	Esophageal cancer	Other malignancies
			(*n* = 401)	(*n* = 144)	(*n* = 92)	(*n* = 78)	(*n* = 65)	(*n* = 22)
Sex								
	Male	*n* (%)	308 (77)	107	74	57	54	16
	Female	*n* (%)	93 (23)	37	18	21	11	6
PS								
	0–1	*n* (%)	375 (94)	139	89	67	61	19
	2	*n* (%)	26 (6)	5	3	11	4	3
Baseline Cr (mg/dL)								
	Median		0.69	0.67	0.68	0.73	0.72	0.71
	(range)		(0.23–1.31)	(0.39–1.11)	(0.24–1.15)	(0.23–1.31)	(0.40–1.10)	(0.49–1.08)
BSA (m^2^)								
	Median		1.61	1.62	1.56	1.60	1.60	1.60
	(range)		(1.15–2.21)	(1.29–2.21)	(1.29–1.96)	(1.22–1.90)	(1.15–1.87)	(1.28–1.92)
BMI (kg/m^2^)								
	Median (range)		21.1 (11.6–35.3)	22.2 (14.9–35.3)	20.9 (11.6–34.0)	20.9 (15.2–33.5)	20.5 (13.4–28.1)	20.6 (16.4–28.8)
Cisplatin dose (mg/m^2^)								
	Median		78.0	78.7	80.0	60.0	70.0	79.8
	(range)		(60.0–105)	(60.0–80.3)	(60.0–105)	(60.0–84.0)	(60.0–80.0)	(60.0–100)
Age (years)								
	Median		65	64	62	67	67	64
	(range)		(28–80)	(33–80)	(30–79)	(28–80)	(51–78)	(37–75)
	≥70	*n* (%)	97 (24)	30	21	25	18	3
	<70	*n* (%)	304 (76)	114	71	53	47	19
Concurrent radiation								
	Yes	*n* (%)	167 (42)	45	60	1	50	11
	No	*n* (%)	234 (58)	99	32	77	15	11
Hypoalbuminemia (serum albumin, <3.0 g/dL)								
	Yes	*n* (%)	43 (11)	14	3	18	8	0
	No	*n* (%)	358 (89)	130	89	60	57	22
Enteral nutrition or TPN								
	Yes	*n* (%)	42 (10)	2	20	2	16	2
	No	*n* (%)	359 (90)	142	72	76	49	20
Type 2 diabetes								
	Yes	*n* (%)	99 (25)	39	30	15	11	4
	No	*n* (%)	302 (75)	105	62	63	54	18
Hydration of ≤2000 mL								
	Yes	*n* (%)	34 (8)	0	23	6	1	4
	No	*n* (%)	367 (92)	144	69	72	64	18
Use of NK1 receptor antagonist								
	Yes	*n* (%)	230 (57)	66	68	46	38	12
	No	*n* (%)	171 (43)	78	24	32	27	10
Intravenous magnesium supplementation								
	Yes	*n* (%)	67 (17)	13	23	16	11	4
	No	*n* (%)	334 (83)	131	69	62	54	18
Oral intake of magnesium oxide as a laxative agent								
	Yes	*n* (%)	164 (41)	56	39	33	28	8
	No	*n* (%)	237 (59)	88	53	45	37	14
Regular use of antihypertensive								
	Yes	*n* (%)	157 (39)	55	44	24	28	6
	No	*n* (%)	244 (61)	89	48	54	37	16
Regular use of NSAIDs								
	Yes	*n* (%)	117 (29)	51	30	18	11	7
	No	*n* (%)	284 (71)	93	62	60	54	15

Drug administration variables refer to the first course of cisplatin chemotherapy. Abbreviations: PS, performance status; Cr, serum creatinine concentration; BSA, body surface area; BMI, body mass index; TPN, total parenteral nutrition; NK1, neurokinin 1; NSAIDs, nonselective nonsteroidal anti-inflammatory drugs.

### Clinicopathologic analysis of risk factors for cisplatin nephrotoxicity

Cisplatin-induced nephrotoxicity was observed in 127 (32%) of the 401 enrolled patients, including 108, 16, and 3 patients with nephrotoxicity of grade 2, 3, and 4, respectively. Among these patients, 55 individuals developed irreversible renal failure. Fisher's exact test revealed that a PS of 2 (*P* = 0.002), the absence of intravenous magnesium supplementation (*P*<0.0001), and the lack of treatment with an NK1 receptor antagonist (*P* = 0.013) were significantly associated with cisplatin nephrotoxicity (Table 2). We also detected significant heterogeneity in the occurrence of nephrotoxicity among tumor types (*P* = 0.045). Examination of the possible impact of concurrent chemotherapy agents on the prevalence of nephrotoxicity (Table S2) revealed no significant association between the use of these agents and such toxicity (*P* = 0.373).

**Table 2:** Comparison of clinicopathologic characteristics as risk factors for cisplatin-induced nephrotoxicity.

Characteristic		Cisplatin nephrotoxicity		*P* value
		Yes (*n* = 127)	No (*n* = 274)	
		*n* (%)	*n* (%)	
Age (years)				
	Median	65	65	0.524
	(range)	(37–80)	(28–80)	
	≥70	31 (32)	66 (68)	0.944
	<70	96 (32)	208 (68)	
Sex				
	Male	97 (31)	211 (69)	0.899
	Female	30 (32)	63 (68)	
PS				
	0 or 1	111 (30)	264 (70)	**0.002**
	2	16 (62)	10 (38)	
Tumor type				
	Lung	40 (28)	104 (72)	**0.045** *
	Head and neck	28 (30)	64 (70)	
	Gastric	23 (29)	55 (71)	
	Esophageal	31 (48)	34 (52)	
	Other	5 (23)	17 (77)	
Concurrent radiation				
	Yes	56 (34)	111 (66)	0.515
	No	71 (30)	163 (70)	
Hypoalbuminemia (serum albumin, <3.0 g/dL)				
	Yes	15 (35)	28 (65)	0.608
	No	112 (31)	246 (69)	
Enteral nutrition or TPN				
	Yes	17 (40)	25 (60)	0.220
	No	110 (31)	249 (69)	
Type 2 diabetes				
	Yes	26 (26)	73 (74)	0.214
	No	101 (33)	201 (67)	
Hydration of ≤2000 mL				
	Yes	13 (38)	21 (62)	0.441
	No	114 (31)	253 (69)	
Use of NK1 receptor antagonist				
	Yes	61 (27)	169 (73)	**0.013**
	No	66 (39)	105 (61)	
Intravenous magnesium supplementation				
	Yes	4 (6)	63 (94)	**<0.0001**
	No	123 (37)	211 (63)	
Oral intake of magnesium oxide as a laxative agent				
	Yes	48 (29)	116 (71)	0.445
	No	79 (33)	158 (67)	
Regular use of antihypertensive				
	Yes	51 (32)	106 (68)	0.826
	No	76 (31)	168 (69)	
Regular use of NSAIDs				
	Yes	44 (38)	73 (62)	0.125
	No	83 (29)	201 (71)	

Drug administration variables refer to the first course of cisplatin chemotherapy. Abbreviations: PS, performance status. TPN, total parenteral nutrition; NK1, neurokinin 1; NSAIDs, nonselective nonsteroidal anti-inflammatory drugs.

*P value for heterogeneity for the occurrence of nephrotoxicity among tumor types. *P* values of <0.05 are shown in bold.

### Multivariable analysis of risk factors for cisplatin nephrotoxicity

To assess the contribution of each individual risk factor to cisplatin-induced nephrotoxicity, we performed multivariable analysis (Table 3). A PS of 2 (risk ratio, 1.876; 95% CI, 1.229–2.864; *P* = 0.004) and regular use of NSAIDs (risk ratio, 1.357; 95% CI, 1.004–1.835; *P* = 0.047) were significantly associated with an increased risk for cisplatin nephrotoxicity, whereas intravenous magnesium supplementation (risk ratio, 0.175; 95% CI, 0.066–0.462; *P* = 0.0004) was associated with a significantly reduced risk. We also found that esophageal cancer was an independent risk factor for nephrotoxicity compared with lung cancer (risk ratio, 1.937; 95% CI, 1.277–2.940; *P* = 0.002). Exploratory analysis revealed no significant interaction between intravenous magnesium supplementation and other covariates (data not shown).

**Table 3:** Risk ratio in multivariable analysis of potential predisposing factors for cisplatin-induced nephrotoxicity (*n* = 401).

Factor		Risk ratio	95% CI	*P* value
Age (≥70 vs. <70 years)		1.006	0.990–1.023	0.475
Sex (male vs. female)		0.947	0.683–1.314	0.745
PS (2 vs. 0 or 1)		1.876	1.229–2.864	**0.004**
Concurrent radiation		1.071	0.769–1.491	0.684
Serum albumin (≥3.0 vs. <3.0 g/dL)		0.897	0.693–1.165	0.419
Enteral nutrition or TPN		0.989	0.643–1.520	0.959
Type 2 diabetes		0.872	0.599–1.270	0.476
Hydration (≤2000 or >2000 mL)		0.801	0.536–1.200	0.283
Use of NK1 receptor antagonist		0.878	0.663–1.163	0.363
Intravenous magnesium supplementation		0.175	0.066–0.462	**0.0004**
Oral intake of magnesium oxide as a laxative agent		0.933	0.703–1.240	0.634
Regular use of antihypertensive		1.010	0.810–1.485	0.553
Regular use of NSAIDs		1.357	1.004–1.835	**0.047**
Tumor type				
	Lung	1.000		
	Head and neck^a^	1.301	0.845–2.010	0.232
	Gastric^a^	1.071	0.678–1.692	0.770
	Esophageal^a^	1.937	1.277–2.940	**0.002**
	Other^a^	0.810	0.360–1.823	0.610

Drug administration variables refer to the first course of cisplatin chemotherapy. Abbreviations: CI, confidence interval; PS, performance status; TPN, total parenteral nutrition; NK1, neurokinin 1; NSAIDs, nonselective nonsteroidal anti-inflammatory drugs.

a These risk factors were compared with lung cancer.

### Effect of magnesium supplementation on serum creatinine levels

**Figure 1:**
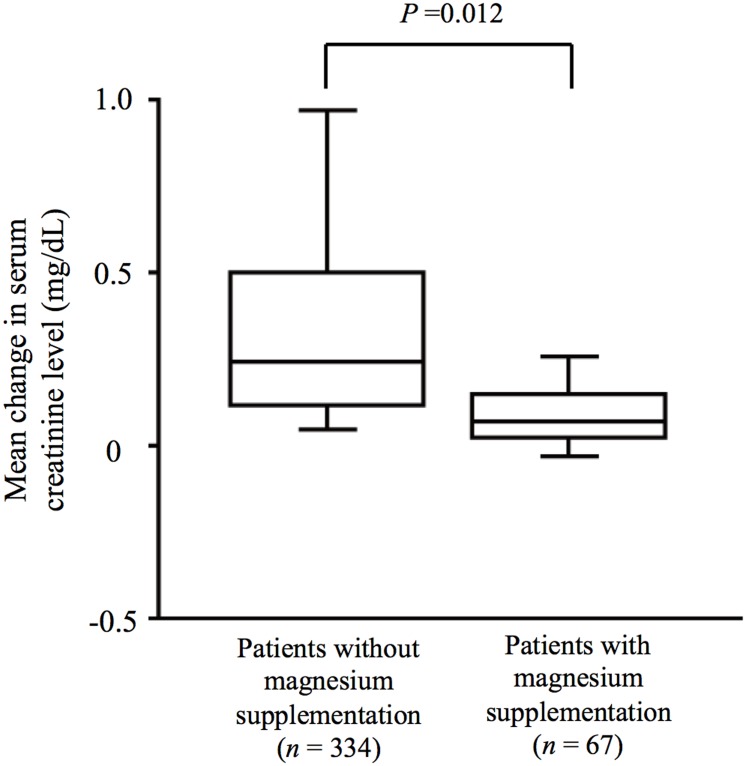
Box-and-whisker plot for the relation between intravenous magnesium supplementation and the mean change in serum creatinine concentration during the first course of cisplatin chemotherapy. The difference between the two groups was analyzed with the unpaired Student's *t* test.

**Figure 2:**
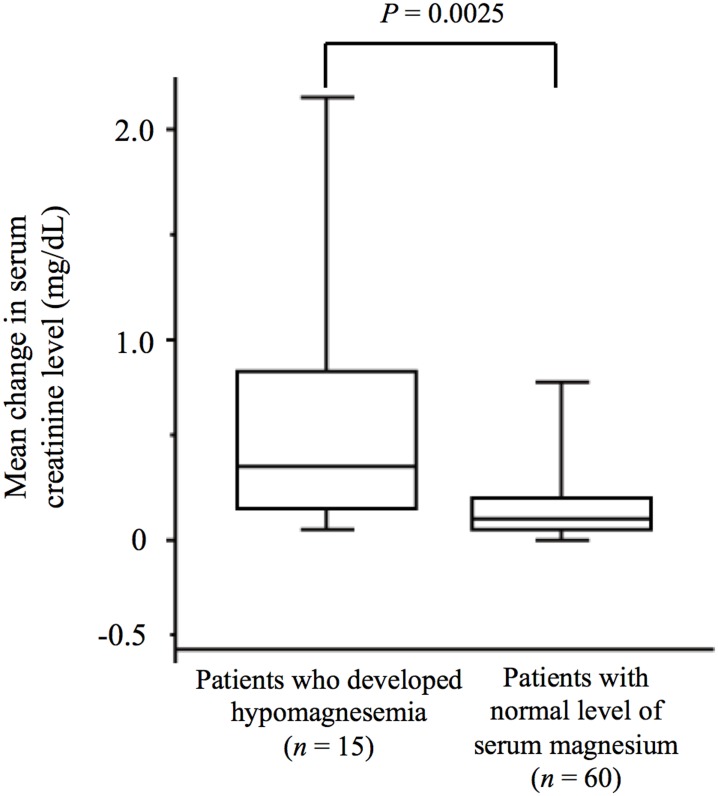
Box-and-whisker plot for the relation between the development of hypomagnesemia and the mean change in serum creatinine concentration during the first course of cisplatin chemotherapy. The difference between the two groups was analyzed with the unpaired Student's *t* test.

As shown in Table 2, we found that the prevalence of cisplatin-induced nephrotoxicity was substantially lower in patients who received intravenous magnesium supplementation than in those who did not (6% vs. 37%). To investigate the effect of magnesium supplementation on cisplatin-induced nephrotoxicity, we evaluated the mean change from baseline in the serum creatinine concentration during the first course of high-dose cisplatin therapy. Patients who received magnesium supplementation therapy (*n* = 67) showed a mean change in serum creatinine level of 0.188±0.081 mg/dL (mean ± SE), whereas those who did not receive the treatment (*n* = 334) showed a mean change of 0.444±0.043 mg/dL (*P* = 0.012), suggesting that magnesium supplementation therapy limited the elevation of serum creatinine level induced by cisplatin (Figure 1). We further examined how magnesium supplementation might prevent cisplatin-induced nephrotoxicity. Data on the serum magnesium concentration during the first course of cisplatin chemotherapy were available for 75 of the 401 study patients. No patient showed hypomagnesemia at baseline. Among the 52 patients who received magnesium supplementation, 6 individuals (12%) developed hypomagnesemia of grade 1 or worse, whereas 9 (39%) of the 23 patients who did not receive magnesium supplementation developed this condition (*P* = 0.040), indicating that magnesium supplementation significantly reduced the proportion of patients who developed hypomagnesemia. Furthermore, the 15 patients who developed hypomagnesemia during cisplatin treatment showed a significantly greater mean increase in the serum creatinine concentration from baseline compared with those who maintained a normal level of serum magnesium (*P* = 0.0025) (Figure 2). These results suggest that intravenous magnesium supplementation protects against cisplatin-induced nephrotoxicity by preventing hypomagnesemia.

## Discussion

Nephrotoxicity remains a clinical problem for 25 to 42% of patients treated with cisplatin [16]–[18]. In the present study, we found that 32% (127/401) of individuals who received cisplatin at a dose of at least 60 mg/m^2^ developed acute nephrotoxicity despite the adoption of conventional measures of hydration and osmotic diuresis. Although the nephrotoxicity was transient and reversible in most cases, 43% (55/127) of the patients with acute nephrotoxicity went on to develop irreversible renal failure. These results indicate that the conventional prophylactic procedures were not sufficient to prevent cisplatin-induced nephrotoxicity in a subset of patients.

We found that magnesium supplementation therapy was significantly associated with both a reduced frequency and reduced severity of renal toxicity, consistent with previous observations [11], [12]. Cisplatin treatment results in a substantial increase in magnesium excretion [19]–[21], with this effect being apparent even before the onset of overt renal toxicity [22], and hypomagnesemia is associated with cisplatin-induced nephrotoxicity [23]. In the present study, a decrease in the serum magnesium concentration was observed in 20% of patients and was significantly associated with renal toxicity during the first course of cisplatin treatment. Organic cation transporter 2 (OCT2) has been implicated in cisplatin nephrotoxicity in a study with isolated human proximal tubules [24], and hypomagnesemia results in up-regulation of OCT2 and thereby increases the renal accumulation of cisplatin and exacerbates acute kidney injury in an animal model [25]. These various findings suggest that magnesium supplementation protects against cisplatin-induced nephrotoxicity, likely by preventing hypomagnesemia, a notion that warrants validation in a prospective study. The dosage of magnesium sulfate for such supplementation therapy has varied widely in previous studies, ranging from 8 to 60 mEq [9]–[11], [13], [26], [27], and it therefore remains to be standardized in future trials.

To assess the potential risk factors for cisplatin-induced nephrotoxicity, we performed multivariable analyses. Consistent with previous results [14], [28], we found that a poor PS was associated with an increased risk for cisplatin nephrotoxicity. This finding underscores the notion that patients with a PS of 2, which is characterized by an increased risk for severe toxicity in general, need special attention with regard to the potential development of nephrotoxicity during high-dose cisplatin chemotherapy, especially given that such treatment in these patients is controversial [29]. We also found that the regular use of NSAIDs was associated with cisplatin-induced nephrotoxicity. Nonselective inhibition of cyclooxygenases 1 and 2 by NSAIDs attenuates prostaglandin-dependent renal function, including modulation of renal vascular tone and electrolyte and water excretion, in particular during renal stress, as manifested by a reduction in the rate of renal perfusion [30], [31]. Such effects of NSAIDs might thus enhance cisplatin-induced nephrotoxicity. Although the significance of the association between the regular use of NSAIDs and cisplatin-induced nephrotoxicity was marginal (*P* = 0.047) in our analysis, it is of concern because NSAIDs are commonly administered to manage cancer-related pain [32]. Further investigations are thus warranted to evaluate the potential risk of regular NSAID use during high-dose cisplatin chemotherapy.

With regard to tumor type, we found that individuals with esophageal cancer were at a significantly higher risk for cisplatin-induced nephrotoxicity than were those with lung cancer. To our knowledge, such an association has not previously been described. The median dosage of cisplatin in patients with esophageal cancer was 70 mg/m^2^, which was not higher than that overall (78 mg/m^2^). Moreover, whereas most patients with esophageal cancer in our analysis were treated with cisplatin together with 5-fluorouracil as the standard care, this regimen was also administered to patients with gastric or head and neck cancer. A difference in dosage or in the combination of chemotherapeutic agents thus could not account for the difference in nephrotoxicity among the malignancies. Caution is necessary in the interpretation of this finding, however, with further study being warranted to determine the mechanism of renal toxicity apparent selectively in patients with esophageal cancer.

Limitations of the present study include possible selection bias of treatment, which is inevitable in a retrospective analysis, and a small sample size for patients with a known serum magnesium concentration and for those who received intravenous magnesium supplementation. Even though all patients treated after July 2011 received magnesium sulfate regardless of their characteristics, cohort effects may still be present that influence the association between magnesium supplementation and nephrotoxicity. In addition, we could not fully assess the incidence and intensity of nonhematologic toxicities in our study as a result of its retrospective nature. Such toxicities, including nausea, vomiting, and diarrhea, might be associated with an increased risk for cisplatin-induced nephrotoxicity. Furthermore, comorbidities relevant to inherent nephrotoxicity, such as proteinuria, hypocalcemia, and renal tubular acidosis, were not assessed in the present study.

In conclusion, our data have revealed a significant association of cisplatin-induced nephrotoxicity with a relatively poor PS and, to a lesser extent, with the regular use of NSAIDs. Our findings also suggest that magnesium supplementation might be effective for protection against the renal toxicity of cisplatin, a conclusion that should be further addressed in a prospective trial.

## Supporting Information

Table S1 **Chemotherapy regimens according to tumor type.**
(DOCX)

Table S2 **Association between concurrent chemotherapy agents and the occurrence of nephrotoxicity.**
(DOCX)
