# Supplementary material for: Risk Factors for Cisplatin-Induced Nephrotoxicity and Potential of Magnesium Supplementation for Renal Protection
Source: PLoS One. 2014 Jul 14;9(7):e101902. doi: 10.1371/journal.pone.0101902 (PMC4096506; doi:10.1371/journal.pone.0101902)
Supplement: Table S1 — Chemotherapy regimens according to tumor type. (DOCX) [file pone.0101902.s001.docx]

**Supplementary Table S1.** Chemotherapy regimens according to tumor type

| Regimen |  | | *n* | % |
| --- | --- | --- | --- | --- |
| Lung cancer (*n* = 144) |  | |  |  |
|  | Cisplatin + vinorelbine | | 42 | 29 |
|  | Cisplatin + vinorelbine + RT | | 23 | 16 |
|  | Cisplatin + etoposide + RT | | 16 | 11 |
|  | Cisplatin + irinotecan | | 16 | 11 |
|  | Cisplatin + pemetrexed | | 16 | 11 |
|  | Cisplatin + etoposide | | 10 | 7 |
|  | Cisplatin + docetaxel | | 9 | 6 |
|  | Cisplatin + S-1 + RT | | 5 | 3 |
|  | Cisplatin + amrubicin | | 4 | 3 |
|  | Cisplatin + irinotecan + RT | | 1 | 1 |
|  | Cisplatin + S-1 | | 1 | 1 |
|  | Cisplatin + gemcitabine | | 1 | 1 |
| Head and neck cancer (*n* = 92) |  | |  |  |
|  | Cisplatin + RT | | 53 | 58 |
|  | Cisplatin + 5-FU | | 30 | 33 |
|  | Cisplatin + 5-FU + RT | | 6 | 7 |
|  | Cisplatin + docetaxel + 5-FU | | 1 | 1 |
|  | Cisplatin + etoposide + RT | | 1 | 1 |
|  | Cisplatin + irinotecan | | 1 | 1 |
| Gastric cancer (*n* = 78) |  | |  |  |
|  | Cisplatin + S-1 | | 51 | 65 |
|  | Cisplatin + capecitabine | | 18 | 23 |
|  | | Cisplatin + irinotecan | 3 | 4 |
|  | | Cisplatin + capecitabine + trastuzumab | 2 | 3 |
|  | | Cisplatin + S-1 + trastuzumab | 2 | 3 |
|  | | Cisplatin + 5-FU + RT | 1 | 1 |
|  | | Cisplatin + 5-FU | 1 | 1 |
| Esophageal cancer (*n* = 65) | |  |  |  |
|  | | Cisplatin + 5-FU + RT | 50 | 77 |
|  | | Cisplatin + 5-FU | 13 | 20 |
|  | | Cisplatin + capecitabine | 1 | 2 |
|  | | Cisplatin + irinotecan | 1 | 2 |
| Other malignancies (*n* = 22) | |  |  |  |
|  | | Cisplatin + RT | 9 | 41 |
|  | | Cisplatin + S-1 | 5 | 23 |
|  | | Cisplatin + S-1 + RT | 1 | 5 |
|  | | Cisplatin + 5-FU + RT | 1 | 5 |
|  | | Cisplatin + 5-FU | 1 | 5 |
|  | | Cisplatin + capecitabine | 1 | 5 |
|  | | Cisplatin + irinotecan | 1 | 5 |
|  | | Cisplatin + doxorubicin | 1 | 5 |
|  | | Cisplatin + etoposide | 1 | 5 |
|  | | Cisplatin + gemcitabine | 1 | 5 |

Abbreviations: RT, concurrent radiation; 5-FU, 5-fluorouracil.
