# Supplementary material for: Risk Factors for Cisplatin-Induced Nephrotoxicity and Potential of Magnesium Supplementation for Renal Protection
Source: PLoS One. 2014 Jul 14;9(7):e101902. doi: 10.1371/journal.pone.0101902 (PMC4096506; doi:10.1371/journal.pone.0101902)
Supplement: Table S2 — Association between concurrent chemotherapy agents and the occurrence of nephrotoxicity. (DOCX) [file pone.0101902.s002.docx]

**Supplementary Table S2.** Association between concurrent chemotherapy agents and the occurrence of nephrotoxicity

| Concurrent agent | | All patients | Positive for nephrotoxicity | |  |
| --- | --- | --- | --- | --- | --- |
|  |  | (*n* = 401) | *n* | % | *P* value* |
| None | | 62 | 23 | 37 | 0.373 |
| Any | | 339 | 104 | 31 |  |
|  | 5-FU | 103 | 38 | 37 |  |
|  | Vinorelbine | 65 | 21 | 32 |  |
|  | S-1 | 63 | 18 | 29 |  |
|  | Etoposide | 28 | 6 | 21 |  |
|  | Irinotecan | 23 | 4 | 17 |  |
|  | Capecitabine | 20 | 6 | 30 |  |
|  | Pemetrexed | 16 | 7 | 44 |  |
|  | Docetaxel | 9 | 2 | 22 |  |
|  | Amrubicin | 4 | 1 | 25 |  |
|  | S-1+ trastuzumab | 2 | 0 | 0 |  |
|  | Gemcitabine | 2 | 0 | 0 |  |
|  | Capecitabine + trastuzumab | 2 | 1 | 50 |  |
|  | Doxorubicin | 1 | 0 | 0 |  |
|  | Docetaxel + 5-FU | 1 | 0 | 0 |  |

Abbreviation: 5-FU, 5-fluorouracil. *Chi-square test.
